# Older age and diclofenac are associated with increased risk of upper gastrointestinal bleeding in gout patients

**DOI:** 10.7717/peerj.11468

**Published:** 2021-05-20

**Authors:** Wan Syamimee Wan Ghazali, Wan Mohd Khairul Bin Wan Zainudin, Nurul Khaiza Yahya, Asmahan Mohamed Ismail, Kah Keng Wong

**Affiliations:** 1 Department of Internal Medicine, School of Medical Sciences, Health Campus, Universiti Sains Malaysia, Kubang Kerian, Kelantan, Malaysia; 2 Department of Immunology, School of Medical Sciences, Health Campus, Universiti Sains Malaysia, Kubang Kerian, Kelantan, Malaysia; 3 Department of Medicine, Hospital Raja Perempuan Zainab II, Kota Bharu, Kelantan, Malaysia

**Keywords:** Gout, Upper gastrointestinal bleeding, Diclofenac

## Abstract

**Background:**

Gouty arthritis is a disease of global burden in which defective metabolism of uric acid causes arthritis. Gouty arthritis or medications used for its treatment may lead to uric acid-associated complications such as upper gastrointestinal bleeding (UGIB) and renal impairment.

**Methods:**

In this cross-sectional study with retrospective record review, 403 established gouty arthritis patients were recruited to determine the incidence of UGIB and associated factors among gout patients who were on regular nonsteroidal anti-inflammatory drugs (NSAIDs).

**Results:**

The mean age of the 403 gouty arthritis patients was 55.7 years old and the majority (*n* = 359/403; 89.1%) were male. The incidence of UGIB among gouty arthritis patients who were on NSAIDs was 7.2% (*n* = 29/403). Older age (*p* < 0.001), diclofenac medication (*p* = 0.003), pantoprazole medication (*p* = 0.003), end-stage renal failure (ESRF) (*p* = 0.007), smoking (*p* = 0.035), hypertension (*p* = 0.042) and creatinine (*p* = 0.045) were significant risk factors for UGIB among the gouty arthritis patients in univariable analysis. Older age (*p* = 0.001) and diclofenac medication (*p* < 0.001) remained significant risk factors for UGIB among the gouty arthritis patients in multivariable analysis.

**Conclusions:**

Age and diclofenac were significantly associated with UGIB among patients with gouty arthritis on regular NSAIDs, indicating that these factors increased the risks of developing UGIB in gout patients. Hence, these high-risk groups of gouty arthritis patients should be routinely monitored to avoid the potential onset of UGIB. Our data also suggest that diclofenac should be prescribed for the shortest duration possible to minimize the risk of developing UGIB in gout patients.

## Introduction

Gout is a disease characterized by defective metabolism of uric acid that leads to arthritis. The incidence and prevalence of gout increase with age, and it is approximately four times more common in men than in women (MacFarlane & Kim, 2014; Ozturk et al., 2013). An elevated serum urate level, together with local factors, can result in the deposition of urate crystals into the joints. Once crystals are deposited into a joint, they can be released into the joint space and initiate an inflammatory cascade causing acute gouty arthritis. These acute flares resolve, but the crystals remain in the joint. The underlying metabolic problem of hyperuricemia and the crystal depositions are treated by lowering the serum urate level and dissolving the crystal deposits. This stops both the acute attacks and the progressive joint damage (Schumacher, 2008).

Clinical and demographic data of gout patients showed that risk factors to develop acute gout were hypertension (53.5%), obesity (40.1%), hyperlipidemia (30.1%), diabetes mellitus (17.9%), and coronary artery disease (17%) (Ozturk et al., 2013). Treatment of gout is centered around resolution of acute attack and preventive measure by reducing uric acid level. In acute setting or acute attacks of gouty arthritis, nonsteroidal anti-inflammatory drug (NSAID) such as diclofenac is the first choice among gout patients. Indomethacin is also a commonly used NSAID for gouty arthritis. Colchicine and prednisolone can also be used for their anti-inflammatory properties that help in reducing pain. Among patients with chronic kidney disease and ischemic heart disease (IHD), the use of NSAIDs is prohibited as it may lead to worsening renal function and heart failure in IHD (Bleumink et al., 2003).

Many gout patients suffer from renal stones, renal failure and upper gastrointestinal bleeding (UGIB) secondary to NSAIDs and gout itself. UGIB is defined as hemorrhage originating from the mouth to duodenum proximal to the ligament of Treitz (Tielleman, Bujanda & Cryer, 2015). It is one of the most common gastrointestinal emergencies, with an average mortality rate of 10%. Despite advances in the diagnosis and management of UGIB, the mortality rate has not changed significantly in the last 50 years (Balaban et al., 2014; Moledina & Komba, 2017). Acute UGIB is a common cause of hospital admission and a leading cause of death in the emergency department. The overall mortality of acute UGIB varies from 3–15% with higher rates of death for those in an unstable hemodynamic state (Tang et al., 2018). UGIB may also be induced by acute gouty arthritis. Continuous bleeding can decrease both blood volume and the glomerular filtration rate, further inducing the reabsorption of uric acid by the proximal convoluted tubule that provokes acute gout (Xu et al., 2015).

The number of patients suffering from UGIB has increased rapidly over recent years due to increased life expectancy and widespread use of drugs such as NSAIDs (Jiang et al., 2015; Kim et al., 2016; Minakari et al., 2017; Petersen et al., 2020). Advanced age has been consistently identified as a risk factor for mortality among patients with UGIB, presumably due to higher prevalence of comorbid illnesses including cardiovascular and pulmonary disease in the elderly (Ahmed & Stanley, 2012). During periods with only NSAID use, the patients demonstrated 3.6 times higher risk to develop UGIB. Concurrent use of corticosteroids, anticoagulants and aspirin further increased the risk of developing UGIB (Mellemkjær et al., 2002).

The association of NSAIDs and UGIB are well-documented. However, there is a lack of documentation on UGIB among gouty arthritis patients on regular NSAIDs. Thus, the aim of this study was to identify the risk factors of UGIB in gout patients who regularly used NSAIDs.

## Materials & methods

### Recruitment of gouty arthritis patients

This was a retrospective study with data of gout patients retrieved from the clinical database of Hospital Raja Perempuan Zainab II in 2018 (1^st^ Jan to 31^st^ Dec 2018). This study was approved by the Human Research Ethics Committee of University Sains Malaysia (ethical approval code: USM/JEPeM/19060379) and Medical Research & Ethics Committee, Ministry of Health Malaysia (ethical approval code: NMRR-19-1575-48858 (IIR)). No informed consent was required due to the retrospective nature of the study where existing human data were used and it was impracticable to obtain individual informed consent. All data were recorded, stored and analyzed anonymously where none of the private information such as name of the patients was disclosed, and a unique identification number instead of patient’s name was used on data collection sheets. All protocols were conducted according to the institutional relevant guidelines and regulations.

Patients were considered eligible for the study if they were above 18 years old, on NSAIDs for 1 year pro re nata and fulfilled criteria from the American College of Rheumatology 2015 classification criteria consistent for gouty arthritis (Neogi et al., 2015). Patients with arthropathy, osteoarthritis, myeloproliferative and lymphoproliferative disorders, HIV positive, malignant diseases and pregnant women were excluded. Patients’ clinico-demographic and laboratory data were obtained from the hospital’s database. All gouty arthritis patients were divided into UGIB or non-UGIB cases. All UGIB cases in this study were endoscopically confirmed, and the event of UGIB was between 1^st^ Jan to 31^st^ Dec 2018. There were no inclusion sub-criteria for hospitalization, hemoglobin drop, hematemesis, melena and other manifestations as the databases were all from inpatient.

The data collected and analyzed were as follows:

1. Demographic data: Age, gender and ethnicity.
2. Clinical data: Smoking history, presence of end-stage renal failure (ESRF), IHD, diabetes mellitus (DM), hypertension or hyperlipidemia, administration of antiplatelet or anticoagulant, NSAIDs and proton pump inhibitors (PPIs).
3. Laboratory results (taken at the time of admission to hospital from inpatient databases): Platelet, creatinine, urea, international normalized ratio (INR) and uric acid.

### Statistical analysis

All statistical analyses were performed using IBM SPSS v22 (SPSS Inc., Chicago, IL, USA). For univariable analysis, two groups of patients with UGIB and non-UGIB were used. Differences between categorical variables were analyzed by chi-squared test or Fisher’s exact test as appropriate, while independent t-test or Mann-Whitney test was used for continuous numerical variables. Binary logistic regression method was used for univariable and multivariable analyses. Any factors whose *p*-value was less than 0.05 in univariable analysis were included in multivariable analysis. For all analyses, a two-tailed *p* < 0.05 was considered as statistically significant.

## Results

### Clinico-demographic and laboratory features

A total of 403 patients were included in this study. The mean age of gouty arthritis patients was 55.7 years old. There were more male (*n* = 359/403; 89.1%) than female patients (*n* = 44/403; 10.9%). This was conducted in Kelantan state of Malaysia where the population is predominantly of ethnic Malay and hence majority of the patients were Malays (*n* = 391/403; 97%) followed by Chinese (*n* = 9/403; 2.2%) and Siamese (*n* = 3/403; 0.7%). Majority of the patients were smokers (*n* = 228/403; 56.6%). Median creatinine level of the study population was 112 µmol, uric acid 582 mmol, platelet 261 × 10^3^ per µl and INR was 1.13 (Table 1).

**Table 1:** Clinico-demographic and laboratory characteristics of the gouty arthritis patients involved in this study (*n* = 403).

Variable		*n* (%)
Age [mean (SD)]		55.7 (16.3)
Sex	Female	44 (10.9)
	Male	359 (89.1)
Race	Malay	391 (97.0)
	Chinese	9 (2.2)
	Siamese	3 (0.7)
Smoking	No	175 (43.4)
	Yes	228 (56.6)
Comorbidities	ESRF	15 (3.7)
	DM	86 (21.3)
	Hypertension	217 (53.8)
	Hyperlipidemia	181 (44.9)
	IHD	94 (23.3)
Laboratory [Mean (SD)]	Creatinine	112 (65.0)*
	Uric Acid	582 (161.0)*
	Platelet	261 (118.0)*
	INR	1.13 (0.24)*
Medication	Aspirin	95 (23.3)
	Clopidogrel	45 (11.2)
	NOAC	14 (3.5)
	Omeprazole	24 (6.0)
	Pantoprazole	34 (8.4)
	Esomeprazole	7 (1.7)
	Ranitidine	30 (7.4)
	Atorvastatin	109 (27.0)
	Simvastatin	48 (11.9)
	Diclofenac	153 (38.0)
	Allopurinol	180 (44.7)
	Colchicine	251 (62.3)
	Prednisolone	75 (18.6)

**Note:**

* Median (interquartile range; IQR).

### Co-morbidities among gouty arthritis patients

Majority of the gouty arthritis patients had hypertension (*n* = 217/403; 53.8%) followed by hyperlipidemia (*n* = 181/403; 44.9%), IHD (*n* = 94/403; 23.3%), DM (*n* = 86/403; 21.3%) and ESRF (*n* = 15/403; 3.7%).

### Medications among gouty arthritis patients

Majority of the study population were prescribed colchicine (*n* = 251/403; 62.3%) and allopurinol (*n* = 180/403; 44.7%). The most common NSAIDs used was diclofenac (*n* = 153/403; 38%) compared with other NSAIDs. A proportion of the patients were on aspirin (*n* = 95/403; 23.3%), clopidogrel (*n* = 45/403; 11.2%) and novel oral anticoagulants (NOACs; consisting of dabigatran, rivaroxaban and apixaban) (*n* = 14/403; 3.5%). Among PPI usage, pantoprazole was the commonest (*n* = 34/403; 8.4%) followed by omeprazole (*n* = 24/403; 6%) and esomeprazole (*n* = 7/403; 1.7%). Ranitidine and prednisolone intake consisted of 34 (8.4%) and 75 (18.6%), respectively. In view of IHD, atorvastatin was prescribed for 109 subjects (27%) compared with simvastatin for 48 patients (11.9%).

### Comparison of clinico-demographic and laboratory features in UGIB and non-UGIB patients

The prevalence of UGIB among gouty arthritis patients in this study population was 7.2% (*n* = 29/403). There were several factors associated with UGIB after comparing each factor with or without UGIB including older age (*p* < 0.001; 67.52 ± 11.84 vs 54.78 ± 16.27 years old), diclofenac medication (*p* = 0.002; *n* = 19/29; 65.5% vs *n* = 134/374; 35.8%), pantoprazole medication (*p*=0.007; n = 7/29; 24.1% vs n = 27/374; 7.2%), creatinine (*p* = 0.008; mean 140 µmol vs mean 127 µmol), ESRF (*p* = 0.017; *n* = 4/29; 13.8% vs *n* = 11/374; 2.9%), non-smokers (*p* = 0.030; *n* = 7/29; 24.1% vs n = 168/374; 44.9%) and hypertension (*p* = 0.037; *n* = 21/29; 72.4% vs *n* = 196/374; 52%). There was no significant association between UGIB and sex, race, co-morbidities (DM, hyperlipidemia, IHD), laboratory parameters (uric acid, platelet, INR) or other drugs (antiplatelet, anticoagulant, NOAC, omeprazole, esomeprazole, ranitidine, allopurinol, colchicine and prednisolone) (Table 2).

**Table 2:** Comparison of characteristics between patients with or without UGIB. *p* < 0.05 is in bold.

Variable	No UGIB (*n* = 374) *n* (%)	UGIB (*n* = 29) *n* (%)	*p*-value
**Age, years [mean (SD)]**	54.78 (16.27)	67.52 (11.84)	**<0.001**^a^
**Sex**			
Female	43 (11.5)	1 (3.4)	0.347^b^
Male	331 (88.5)	28 (96.6)	
**Race**			
Malay	364 (97.3)	27 (93.1)	0.122^b^
Chinese	8 (2.1)	1 (3.4)	
Siamese	2 (0.5)	1 (3.4)	
**Smoking**			
No	168 (44.9)	7 (24.1)	**0.030**^c^
Yes	206 (55.1)	22 (75.9)	
**Comorbidities**			
ESRF	11 (2.9)	4 (13.8)	**0.017^b^**
DM	78 (20.9)	8 (27.6)	0.394^c^
Hypertension	196 (52.4)	21 (72.4)	**0.037**^c^
Hyperlipidemia	167 (44.7)	14 (48.3)	0.705^c^
IHD	84 (22.5)	10 (34.5)	0.140^c^
**Creatinine, median (IQR)**	127.0 (71.0)	140.0 (160.0)	**0.008^d^**
**Uric acid, median (IQR)**	586.0 (168.0)	544.0 (146.0)	0.921^d^
**Platelet, median (IQR)**	260.0 (120.0)	261.0 (105.0)	0.988^d^
**INR, median (IQR)**	1.12 (0.28)	1.27 (0.25)	0.172^d^
**Medication**			
**Antiplatelet/Anticoagulant**			
Cardiprin	84 (22.5)	11 (37.9)	0.059^c^
Clopidogrel	43 (11.5)	2 (6.9)	0.758^b^
NOAC	14 (3.7)	0 (0.0)	0.612^b^
**PPI**			
Omeprazole	23 (6.1)	1 (3.4)	>0.950^b^
Pantoprazole	27 (7.2)	7 (24.1)	**0.007**^b^
Esomeprazole	7 (1.9)	0 (0.0)	>0.950^b^
Ranitidine	29 (7.8)	1 (3.4)	0.712^b^
**Anti lipids**			
Atorvastatin	101 (27.0)	8 (27.6)	>0.950^c^
Simvastatin	46 (12.3)	2 (6.9)	0.556^b^
**NSAIDs/Anti-gout**			
Diclofenac	134 (35.8)	19 (65.5)	>**0.002^c^**
Allopurinol	163 (43.6)	17 (58.6)	0.117^c^
Colcichine	231 (61.8)	20 (69.0)	0.441^c^
Prednisolone	73 (19.5)	2 (6.9)	0.092^c^

**Notes:**

a Independent sample t-test;

b Fisher’s exact test;

c Chi-squared test;

d Mann-Whitney test.

### Risk factors for UGIB in gouty arthritis patients

In terms of univariable logistic regression analysis, five categorical variables i.e., diclofenac (*p* = 0.003), pantoprazole (*p* = 0.003), ESRF (*p* = 0.007), smoking (*p* = 0.035) and hypertension (*p* = 0.042), and 2 numerical variables i.e., age (*p* < 0.001) and creatinine (*p* = 0.045) were significant risk factors for UGIB in gout patients (Table 3). These seven variables were subsequently selected for multivariable analysis. Older age (HR: 1.06; 95% CI: 1.02–1.09; *p* = 0.001) and diclofenac (HR: 5.29; 95% CI: 2.22–12.60; *p* < 0.001) remained as significant risk factors for UGIB in gout patients (Table 4). The rest of the variables were not significantly associated with UGIB i.e., pantoprazole (*p* = 0.109), ESRF comorbidity (*p* = 0.341), hypertension (*p* = 0.519), higher creatinine (*p* = 0.728) and smoking (*p* = 0.921) (Table 4).

**Table 3:** Risk factors associated with UGIB in gout patients according to univariable logistic regression analysis. *p* < 0.05 is in bold.

Risk factor	HR [95% CI]	*p*-value
**Age**	1.06 [1.03–1.09]	**<0.001**
**Sex**	0.28 [0.04–2.07]	0.210
**Race**	0.25 [0.01–5.99]	0.392
**Smoking**	2.56 [1.07–6.15]	**0.035**
**ESRF**	5.28 [1.57–17.78]	**0.007**
**DM**	1.45 [0.62–3.39]	0.396
**Hypertension**	2.38 [1.03–5.52]	**0.042**
**Hyperlipidemia**	1.16 [0.54–2.47]	0.706
**IHD**	1.82 [0.81–4.06]	0.145
**Creatinine**	1.00 [1.00–1.00]	**0.045**
**Uric acid**	2.11 [0.96–4.64]	0.063
**Platelet**	1.00 [1.00–1.00]	0.568
**INR**	1.85 [0.46–7.50]	0.389
**Cardiprin**	2.11 [0.96–4.64]	0.063
**Clopidogrel**	0.57 [0.13–2.48]	0.454
**NOAC**	0.00 [0.00–0.00]	0.999
**Omeprazole**	0.55 [0.07–4.19]	0.560
**Pantoprazole**	4.09 [1.60–10.43]	**0.003**
**Esomeprazole**	0.00 [0.00–0.00]	0.999
**Ranitidine**	0.43 [0.06–3.23]	0.409
**Atorvastatin**	1.03 [0.44–2.40]	0.946
**Simvastatin**	0.53 [0.12–2.30]	0.394
**Diclofenac**	3.40 [1.54–7.53]	**0.003**
**Allopurinol**	1.83 [0.85–3.95]	0.121
**Colcichine**	1.38 [0.61–3.10]	0.442
**Prednisolone**	0.31 [0.07–1.31]	0.111

**Table 4:** Risk factors associated with UGIB in gout patients according to multivariable logistic regression analysis. *p* < 0.05 is in bold.

Risk factor	HR [95% CI]	*p*-value
**Diclofenac**	5.29 [2.22–12.60]	**<0.001**
**Older age**	1.06 [1.02–1.09]	**0.001**
**Pantoprazole**	2.28 [0.83–6.28]	0.109
**ESRF comorbidity**	3.25 [0.29–36.81]	0.341
**Hypertension**	0.73 [0.28–1.91]	0.519
**Higher creatinine**	1.00 [1.00–1.00]	0.728
**Smoking**	1.05 [0.39–2.84]	0.921

## Discussion

The incidence of UGIB associated with NSAIDs is well-documented but the incidence of this association among gouty arthritis patients has not been addressed. To the best of our knowledge, this study is the first to report the incidence of UGIB among gouty arthritis and its associated factors.

Our cohort of patients involved more males than females with gouty arthritis. An independent meta-analysis showed that men were prone to gout with four-fold higher incidence than women but the incidence reduced to 3:1 ratio for patients over 65 years old (Wallace et al., 2004). It has been reported that post-menopausal females are more prone to be diagnosed with UGIB than males (Mellemkjær et al., 2002) likely due to lack of estrogen’s protective effect on the gastric mucosal layer.

Patients with long-term use of NSAIDs have higher occurrence of peptic ulcer (Schoenfeld et al., 1999), renal impairment and increased cardiovascular events whereby diclofenac has been reported to be the highest risk factor associated with major cardiovascular events (Nissen et al., 2016; Trelle et al., 2011). This led to recommendations against the use of NSAIDs for treatment of gout flares in patients with severe cardiovascular disease or late stages of chronic kidney disease. If NSAIDs must be used, they should only be prescribed at the time of the flare according to the 2020 recommendations from the French Society of Rheumatology for the management of gout (Latourte et al., 2020). Essentially, usage of NSAIDs is a common causative factor of UGIB (Coxib et al., 2013; Gralnek, Barkun & Bardou, 2008), and elderly patients on NSAIDs are the most vulnerable group of patients to develop UGIB (Peiro Moreno et al., 2011; Sostres & Lanas, 2011) where NSAID induced UGIB in nearly half of elderly patients (Leung Ki & Chan, 2012). Collectively, our data support the proposal of diclofenac medication for the shortest duration possible, that is at the time of gout flares only, in order to reduce the risk of diclofenac-induced side effects including UGIB.

Our cohort of gouty arthritis patients on diclofenac demonstrated 5.29 times higher risk to have UGIB compared to those not on diclofenac. A meta-analysis showed that a positive relationship between diclofenac dose and with increased risk for UGIB or cardiovascular event compared with other NSAIDs (Odom et al., 2014). To the best of our knowledge, there is no previous data pertaining to incidence of GI bleeding secondary to diclofenac use due to other causes. However, there are multiple risks factors that may contribute to UGIB in patients on diclofenac regardless of presenting with gout or not. These risk factors include advanced age, previous history of GI bleeding, and concurrent use of medications such as anticoagulants, aspirin, corticosteroids and selective serotonin reuptake inhibitors (Odom et al., 2014). Antiplatelet and anticoagulant medications are known risk factors that cause UGIB. However, there was no significant difference in our cohort of UGIB vs non-UGIB patients in terms of antiplatelet or anticoagulant usage. This may be due to the fact that our patients being on concurrent PPI medication that could contribute to gastroprotection. In addition, PPIs are protective medication against UGIB but gout patients that took pantoprazole had significant cause of UGIB in this study by univariable analysis. However, it was not known whether the subjects had taken pantoprazole after or prior to UGIB events.

We acknowledge the limitations of this study as follows: (1) Diclofenac was the only NSAID included in our analysis due to the number of patients receiving other NSAIDs such as naproxen, etoricoxib, indomethacin or celecoxib was insufficient for inclusion in the statistical analyses. We recommend further studies involving multiple types of NSAIDs including their frequency and dosage; (2) The retrospective nature of our study and the lack of clinical details about the severity, types and outcomes of UGIB; (3) Our simple binary outcome of UGIB or non-UGIB is less interesting to clinicians when compared to other UGIB outcomes such as Glasgow-Blatchford or AIMS65 bleeding score, transfusion requirements, need for intensive care unit admission or endoscopic intervention, and the length of hospital stay.

## Conclusions

In conclusion, older age and diclofenac prescription were significant risk factors for UGIB development in gouty arthritis patients on NSAIDs. Thus, patients presented with these risk factors should be routinely monitored to prevent the occurrence of UGIB. We also recommend that diclofenac be prescribed for the shortest duration possible to manage gout flares.

## Supplemental Information

10.7717/peerj.11468/supp-1 Supplemental Information 1 Raw data of the demographic, clinical and laboratory parameters of all 403 gout patients involved in this study.

## Additional Information and Declarations

### Data Availability

The following information was supplied regarding data availability: The raw data is available in the Supplemental File.
